# Association between low potassium intake and the number of teeth in Korean adults: based on the national data (2013–2015)

**DOI:** 10.1038/s41598-021-82631-4

**Published:** 2021-02-04

**Authors:** Eun-Jeong Kim, Hye-Ju Lee

**Affiliations:** 1 Department of Dental Hygiene, Gangdong University, Chungcheong, Korea; 2 Dental Research Institute, Seoul National University, Seoul, Korea; 3 Department of Dental Hygiene, College of Health Science, Sun Moon University, Asan, Korea

**Keywords:** Health care, Risk factors

## Abstract

General health and oral health are very closely related. This study aimed to analyze the nutritional factors associated with the number of present teeth in Korean elderly adults. A total of 6,356 individuals were surveyed from the sixth Korean National Health and Nutrition Examination Survey conducted from 2013–2015. The number of existing teeth was divided into three categories: 0–10, 11–20, and over 21, and the nutrition survey covers eating habits, food frequency and food intake using face-to-face interviews. Multiple logistic regression analysis was performed to evaluate the association between nutrient intake and the number of existing teeth after adjusting for socio-demographic factors and general and oral health behaviors and status. As age increases, the number of teeth decreases. Individuals with more teeth had a significantly higher mean daily intake of protein, calcium, phosphorus, potassium and riboflavin (p < 0.05). After adjusting for sociodemographic factors in model 1 and the lower number of teeth in model 2, the strength of the association between the number of teeth and daily calcium intake remained significant. Statistically significant associations were present for dietary potassium intake in models 1 and 2 and in the 11–20 teeth group in model 3 (p < 0.05). We demonstrated a significant association between calcium and potassium intake and the number of teeth.

## Introduction

General health and oral health are very closely related. As life expectancy has been extended, recent attention is focused on oral health in relation to overall well-being. The welfare of nutrition plays an essential role in improving and maintaining the health of the elderly^1^. According to the recent study, oral health has been associated to the amount and quality of food consumed^2^. In addition, it was reported that a poor oral health condition and a low intake of a specific food were related^3–7^.

Tooth loss due to oral health leads to a decrease in chewing ability and it can limit a person’s diet to one of bad nutritional qualities and affect nutritional status. So it could affect the general health for many people^8^. Older people with fewer teeth are vulnerable to dietary restrictions. Loss of teeth is linked to a change in the food preferences of older people and a lack of nutrition^9–11^. Several studies^7,12,13^ have reported that the number of teeth and the intake of adequate nutrients play a similar role in eating fruits and vegetables, such as reducing the risk of chronic diseases such as cardiovascular disease and high blood pressure.

Analysis of the effects of tooth loss on nutrient intake also showed that dietary fiber, carotene, fruits and vegetables have decreased as natural teeth decrease, while the average intake of calories and saturated fats and cholesterol tends to increase with the decrease in the number of teeth^14^. In addition, blood pressure and periodontal disease increased due to decrease in potassium intake accompanying dietary fiber intake, and this results can be inferred that blood pressure and periodontal disease may decrease by increasing potassium consumption^15^. However, the association between poor oral health and certain levels of nutrition is not well documented.

So far, there have been cases in which national research has conducted and reported dietary patterns for the elderly or dietary analysis related to oral disease, while there have been studies focusing on calcium, phosphorus and vitamins in the relationship between existing number of teeth and nutrients, but there are very few research reports on minerals including potassium reported to be closely related to periodontal disease.

Therefore, the aim of this study was to analyze the nutritional factors associated with the number of present teeth in Korean elderly adults aged 55–84 years, considering the effect of modifiers, among a representative sample of Korean adults, after controlling for age, sex, household income, education, use of dental floss, use of interproximal brush, periodontitis, alcohol drinking, smoking, hypertension, diabetes and obesity.

## Materials and methods

### Study design and subject selection

**Figure 1:**
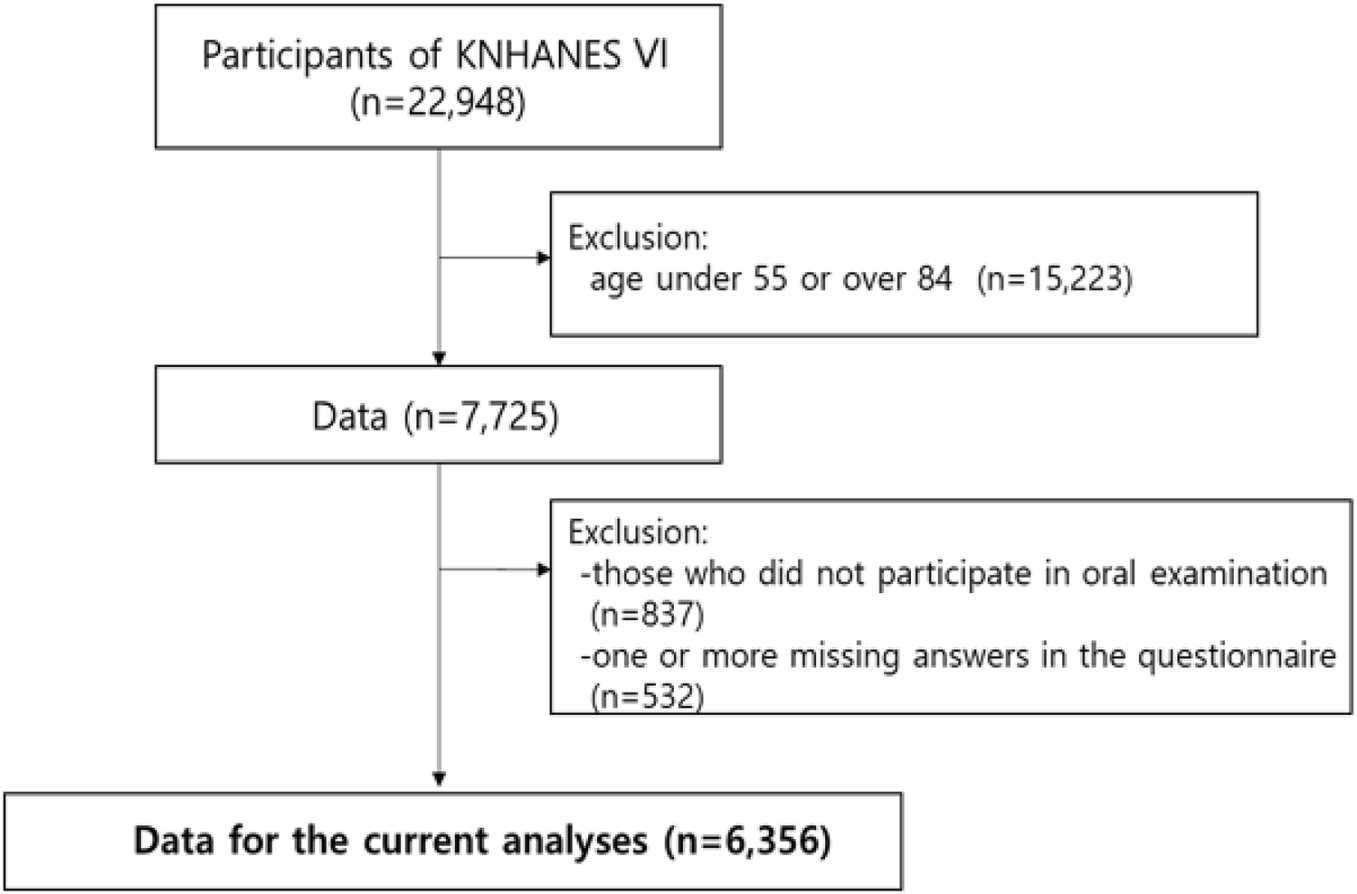
Flow chart of study selection.

The study data include a partial set of the sixth Korea National Health and Nutrition Examination Survey (KNHANES), conducted between 2013 to 2015 by the Korea Centers for Disease Control and Prevention (KCDCP). The KNHANES identifies the current status and trends of the health and nutritional status of the people, selects health vulnerable groups to be given policy priorities, and calculates statistics necessary to evaluate whether health policies and projects are being effectively delivered. In addition, the survey is conducted to provide statistical data related to smoking, drinking, physical activity, and obesity requested by the World Health Organization (WHO) and the Organization for Economic Cooperation and Development (OECD)^16^. The KNHANES sampling protocol is designed to include a complex, layered multi-layer and probability cluster survey of representative samples of the uninstructed private population in Korea. The survey was conducted by Korea’s Ministry of Health and Welfare. The survey included all those aged 1 and older. The survey used stratified multistage probability sampling units according to geographical region, gender and age, which were determined based on the household registry of the National population survey register of 2010, the latest 5-year national census in Korea. Using the 2010 census data, 576 primary sampling units (PSU) were selected across Korea. The final sample of KNHANES included 11,520 households. It included highly structured health-related questionnaires, a nutrition survey and an oral health examination conducted by trained dentists. Each participant in the survey signed an informed consent form. A detailed description of the sampling can be found in the KNHANES report^16^. The total number of participants in KNHANES VI was 22,948. The study’s age group 55–84 years. The exclusion criteria were (i) those who did not participate in oral examination, and (ii) one or more missing answers in the questionnaire. The final total sample size for the fully adjusted model was 6,356 (Fig. 1). The KCDCP institutional review board approved KNHANES (2013-07CON-03-4C, 2013-12EXP-03-5C, and 2015-01-02-6C) and all subjects received ethical approval and informed consent. All methods were carried out in accordance with relevant guideline and regulations.

### Oral examination and clinical variables

Oral health examinations were conducted by dentists. The calibration was performed in the clinical examinations with the subject seated in the dental chair using a light, a mouth mirror and a WHO periodontal probe. The WHO criteria were used to assess oral health status^17^. We measured the number of teeth in the sample subjects, excluding missing teeth, impacted teeth or implants, and third molars. The root tips and teeth marked for extraction were also classified as missing teeth. The number of teeth totaled 28, excluding the third molar. The number of existing teeth was divided into three categories: 0 to 10, 11 to 20, and over 21^18^.

### Dietary intake

The nutrition survey includes the contents on food and food intake frequency and intake by food item^19^. The food frequency questionnaire consists of 63 food items that refer to the main sources of energy and nutrients. Food intake questionnaires are designed with an open survey to report a variety of foods and using the 24-h Single Diet Recall (DR) method with various measurement aids. Food intake, energy, carbohydrate, fat, protein, calcium, phosphorus, potassium, vitamin C and riboflavin intake for each respondent were computed using the food composition table produced by the Korea National Rural Resources Development Institute^20,21^. A professional research team consisting of 4 teams of 2 each visits the household and conducts a computer-assisted personal interview (CAPI). This is the only survey that directly and continuously surveys the Korean people's food intake by individuals, and it is very accurate because the actual cooked food recipe is directly surveyed and used for analysis.

### Assessment of confounders

Demographics included age, gender, household income and education, dental floss use, oral health behaviors such as brushing brush use and periodontitis, behavioral factors such as drinking and smoking, and systemic factors such as diabetes, high blood pressure and obesity. In an interview, socio- demographic, oral health behavior and behavioral factors were surveyed. General health-related factors were evaluated in questionnaires, clinical examinations and laboratory procedures. Age was divided into three groups: 55–64, 65–74, and 75–84. Monthly household income was adjusted to the number of families and classified into four deciles from top to bottom. The level of education was divided into four groups: elementary, middle, high school and college. The smoking status was divided into three categories (currently vs. stop vs. never). The frequency of alcohol consumption was divided into two groups: non-drinkers and drinkers. High blood pressure is caused by people with systolic blood pressure above 140 mmHg or above 90 mmHg, or those who take drugs with high blood pressure. Diabetes was defined as having fast glucose levels above 126 hg/dL or taking drugs for diabetes. Obesity was classified into three states according to the body mass index (BMI): normal for18.5 kg/m^2^ < BMI < 25 kg/m^2^, underweight for BMI < 18.5 kg/m^2^, and obese for BMI > 25 kg/m^2^. The use of flossing and interproximal brushes is grouped by yes or no. The periodontal state was carefully assessed for the quantification of periodontitis, and Community Periodontal Index of Treatment Needs (CPITN) was used to quantify periodontitis. The selected teeth were 11, 16, 17, 26, 27, 31, 36, 37, 46 and 47. The Community Periodontal Index (CPI) was scored from 0 to 4, 0 (normal), 1 (gingivitis with bleeding during probing), 2 (presence of calculus), 3 (pocket depth ≤ 3.5 mm) and 4 (pocket depth ≤ 5.5 mm). The periodontal condition was classified into two categories: absence of periodontitis (CPI 1–2) and presence (CPI 3–4).

### Statistical analysis

Individual weighted factors were used, and the complex sampling design of the survey was considered to obtain the variances. To evaluate combined effects of tooth loss and nutrient intake, we created a categorical variable for teeth: 0–11, 11–20 and over 21 teeth. For the characteristics of the participants by number of existing teeth, chi-square tests of complex sample analysis with weight application were performed to estimate the weighted proportions (95% confidence interval [CI]) of the total population sample.

Multiple logistic regression analysis of complex sample analysis with weight application was performed to evaluate the adjusted association between nutrient intake and number of existing teeth. Model 1 represents a crude association, while model 2 is adjusted for sociodemographic factors. Model 3 is adjusted for all the variables in model 2, including oral health status and behaviors, and model 4 is adjusted for all the variables in model 3, including general health status and behaviors.

To account for the complex survey design of stratified, random and cluster sampling, all statistical analyses were performed using SPSS Complex Samples Procedures (IBM SPSS statistics 21, IBM Inc., Chicago, IL, USA). The values of p < 0.05 were considered statistically significant.

## Results

Table 1 lists the characteristics of the study subjects categorized by the number of existing teeth. Of the 6356 participants included in the analyses, 1289 participants have 0–11 teeth, 1434 have 11–20 teeth and 3633 have over 21 teeth in our data. As the age increases, the number of teeth decreases. Monthly household income, education, smoking status, alcohol consumption, hypertension diabetes mellitus, obesity, use of floss, use of interproximal tooth brush and periodontitis were significantly different based on the number of existing teeth.

**Table 1:** Characteristics of the participants according to number of teeth group (N = 6356).

Characteristic	Number	0–10 teeth		11–20 teeth		21 + teeth		*p* value
		N	% (95% CI)*	N	% (95% CI)*	N	% (95% CI)*	
**Age**								** < 0.001**
55–64	2596	187	18.0 (15.5–20.8)	436	35.6 (32.4–39.0)	1973	61.1 (59.0–63.2)	
65–74	2363	495	37.9 (35.0–40.9)	635	41.8 (38.8–44.9)	1233	29.2 (27.5–31.0)	
75–84	1397	607	44.1 (41.1–47.1)	363	22.6 (20.3–25.0)	427	9.1 (8.6–10.9)	
**Sex**								0.124
Male	2698	572	46.8 (43.7–49.8)	629	47.6 (44.6–50.7)	1497	44.3 (42.6–45.9)	
Female	3658	717	53.2 (50.2–56.3)	805	52.4 (49.3–55.4)	2136	55.7 (54.1–57.4)	
**Monthly household income**								** < 0.001**
< 25%	2286	717	53.7 (50.0–57.3)	574	37.8 (34.6–41.1)	995	24.6 (22.7–26.6)	
25–50%	1707	303	23.9 (21.1–27.0)	417	29.1 (26.2–32.2)	987	26.6 (25.0–28.3)	
50–75%	1227	162	14.5 (12.0–17.3)	265	20.2 (17.7–22.8)	800	23.6 (21.8–25.5)	
> 75%	1088	90	8.0 (6.3–9.9)	167	12.9 (10.8–15.3)	831	25.3 (23.0–27.7)	
**Education**								** < 0.001**
Primary school	2922	771	66.3 (62.9–69.6)	725	52.5 (49.0–55.9)	1426	38.1 (35.8–40.5)	
Middle school	982	140	13.6 (11.4–16.1)	223	17.9 (15.6–20.4)	619	19.1 (17.4–20.9)	
High school	1217	154	14.8 (12.5–17.4)	255	20.6 (18.0–23.5)	808	26.0 (24.2–28.0)	
College	694	55	5.3 (4.0–7.2)	101	9.0 (7.1–11.4)	538	16.7 (14.8–18.9)	
**Smoking status**								** < 0.001**
Current	775	212	18.3 (15.9–21.0)	191	16.1 (13.8–18.8)	372	11.8 (10.6–13.3)	
Stop	1529	310	25.6 (23.0–28.3)	362	26.7 (24.1–29.5)	857	25.1 (23.5–26.7)	
Never	3908	725	56.1 (53.2–59.0)	847	57.1 (54.0–60.3)	2336	63.1 (59.2–61.7)	
**Alcohol consumption**								** < 0.001**
No	1326	334	25.2 (22.5–28.1)	325	21.9 (19.4–24.6)	667	16.9 (15.6–18.3)	
Yes	4886	913	74.8 (71.9–77.5)	1075	78.1 (75.4–80.6)	2898	83.1 (81.7–84.4)	
**Hypertension**								** < 0.001**
No	3472	647	52.6 (49.2–56.0)	718	52.7 (49.6–55.7)	2107	60.7 (58.9–62.5)	
Yes	2884	642	47.4 (44.0–50.8)	716	47.3 (44.3–50.4)	1526	398.3 (37.5–41.1)	
**Diabetes mellitus**								** < 0.001**
No	5259	1016	79.3 (76.4–81.8)	1155	79.8 (77.2–82.2)	3088	85.7 (84.5–86.9)	
Yes	1097	273	20.7 (18.2–23.6)	279	20.2 (17.8–22.8)	545	14.3 (13.1–15.5)	
**Obesity**								** < 0.001**
Underweight	173	57	4.2 (3.0–5.6)	42	2.8 (2.0–3.9)	74	1.9 (1.4–2.5)	
Normal	3839	812	63.8 (60.6–66.8)	833	58.0 (55.1–60.9)	2194	61.1 (59.2–63.0)	
Obese	2332	415	32.1 (29.2–35.1)	556	39.2 (36.3–42.2)	1361	37.0 (35.1–38.9)	
**Use of floss**								** < 0.001**
No	5545	1215	97.5 (96.2–98.3)	1298	93.1 (91.5–94.5)	3032	85.2 (83.7–86.5)	
Yes	664	31	2.5 (1.7–3.8)	101	6.9 (5.5–8.5)	532	14.8 (13.5–16.3)	
**Use of interproximal tooth brush**								** < 0.001**
No	5379	1158	92.3 (90.4–93.8)	1216	86.4 (84.0–88.5)	3005	83.8 (82.2–85.2)	
Yes	830	88	7.7 (6.2–9.6)	183	13.6 (11.5–16.0)	559	16.2 (14.8–17.8)	
**Periodontitis**								** < 0.001**
No	3130	492	64.5 (60.2–68.5)	688	48.1 (44.6–51.7)	1950	53.3 (50.8–55.7)	
Yes	2667	258	35.5 (31.5–39.8)	733	51.9 (48.3–55.4)	1676	46.7 (44.3–49.2)	

Values are presented as number (%). Monthly Household income: monthly average family equivalent income (-monthly average household income/√(the number of household members). Obesity; Normal; Body Mass Index (BMI) 18.5 to < 25, Underweight: BMI < 18.5, and Obesity: BMI ≥ 25.0 kg/m^2^. Periodontitis was defined as community periodontal index 3–4.

*Weighted percent, 95% Confidence Interval (CI), and p-value obtained by Chi-square test.

The results of the complex samples general linear model are shown in Table 2. In Table 2, after adjusting for age, sex, household income, smoking status, alcohol consumption, use of floss, use of interproximal brush, hypertension, obesity and diabetes, those with more teeth had a significantly higher mean daily intake of protein, calcium, phosphorus, potassium and riboflavin (p < 0.05).

**Table 2:** Adjusted geometric means of daily nutrient intake by number of existing teeth*.

Nutrients	0–10 teeth (n = 6356)		11–20 teeth (n = 1434)		< 21 teeth (n = 3633)		*p*
	M^†^	95% CI	M^†^	95% CI	M^†^	95% CI	
Food intake	1326.75	1250.59–1402.91	1347.63	1273.94–1421.33	1387.47	1316.54–1458.39	0.097
Energy	1717.18	1642.38–1791.98	1763.34	1689.65–1837.03	1768.41	1700.23–1836.58	0.199
Carbohydrate	289.89	276.51–303.28	298.34	285.68–311.01	297.05	285.57–308.53	0.284
Fat	29.83	27.48–32.17	29.96	27.61–32.31	31.74	29.42–34.06	0.094
Protein	56.06	53.00–59.11	57.82	54.85–60.79	59.52	56.58–62.47	**0.016**
Calcium	400.84	372.16–429.53	435.35	406.14–464.57	447.56	422.33–472.78	** < 0.001**
Phosphorus	922.02	875.26–968.79	958.02	912.99–1003.05	981.24	938.23–1024.25	**0.003**
Potassium	2775.7	2605.90–2945.49	2823.55	2667.98–2979.11	2946.76	2798.34–3095.17	**0.02**
Vitamin C	110.47	96.90–124.05	105.98	93.71–118.26	116.62	105.28–127.96	0.088
Riboflavin	1.07	1.00–1.14	1.08	1.01–1.15	1.15	1.08–1.21	**0.004**

The data was analyzed by complex samples general linear model. Bold values denotes statistical significance at p < 0.05.

*Adjusted for age, sex, household income, smoking status, alcohol consumption, use of floss, use of interproximal brush, hypertension, obesity, and diabetes.

^†^Geometric mean.

**Figure 2:**
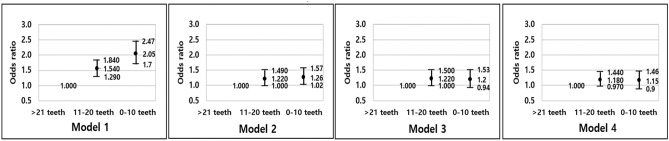
Adjusted association of daily potassium intake with number of teeth. Model 1 was unadjusted association, model 2 was adjusted for age, sex, household income and education, model 3 was adjusted for all variables in model 2 and use of floss, use of interproximal brush and periodontitis, and model 4 was adjusted for all variables in model 3 and smoking status, alcohol consumption, hypertension, diabetes mellitus and obesity. Diamond denotes and bar denotes 95% confidence interval. Diamond with value 1 represents the reference.

The number of teeth was found to be associated with dietary calcium intake and potassium intake (Table 3, Fig. 2). After adjusting for sociodemographic factors in model 1 and the 0–10, and 11–20 teeth in model 2, the strength of the association between the number of teeth and daily calcium intake remained significant. Statistically significant associations were present for dietary potassium intake in models 1 and 2 and in the 11–20 teeth group in model 3 (p < 0.05).

**Table 3:** Adjusted association between the number of existing permanent teeth and dietary calcium and potassium intake (within recommendation and below recommendation) (n = 6,356).

Variable	The number of existing teeth	Number	OR (95% CI)			
			Model 1^a^	Model 2^b^	Model 3^c^	Model 4^d^
**Daily calcium intake**						
	0–10 teeth	1289	**2.28 (1.81–2.86)**	**1.53 (1.17–1.99)**	1.33 (0.97–1.82)	1.32 (0.97–1.81)
	11–20 teeth	1434	**1.42 (1.15–1.76)**	1.26 (0.98–1.60)	1.26 (0.99–1.61)	1.25 (0.97–1.60)
	< 21 teeth	3633	1	1	1	1
**Daily potassium intake**						
	0–10 teeth	1289	**2.05 (1.70–2.47)**	**1.26 (1.02–1.57)**	1.20 (0.94–1.53)	1.15 (0.90–1.46)
	11–20 teeth	1434	**1.54 (1.29–1.84)**	**1.22 (1.00–1.49)**	**1.22 (1.00–1.50)**	1.18 (0.97–1.44)
	< 21 teeth	3633	1	1	1	1

The dependent variable was daily calcium and potassium intake.

*OR* odds ratio, *CI* confidence interval. Below recommendation of calcium was < 700 mg, within recommendation of calcium was ≥ 700 mg. Below recommendation of potassium was < 3500 mg, within recommendation of potassium was ≥ 3500 mg.

^a^Model 1 was unadjusted association.

^b^Model 2 was adjusted for age, sex, household income and education.

^c^Model 3 was adjusted for all variables in model 2 and use of floss, use of interproximal brush and periodontitis.

^d^Model 4 was adjusted for all variables in model 3 and smoking status, alcohol consumption, hypertension, diabetes mellitus and obesity.

Bold values denotes statistical significance at p < 0.05.

## Discussion

As the age group increased, the number of existing permanent teeth decreased. As a result of complex samples general linear analyses of nutrient intake and existing teeth after adjusting for age, sex, household income, smoking status, alcohol consumption, use of floss, interproximal brush, hypertension, obesity, and diabetes, the consumption of nutrients such as protein, calcium, phosphorus, potassium, riboflavin decreased in the group with fewer teeth. This finding was consistent with those of the previous study, which reported on the relationship among dental status, nutrient intake, and nutritional status in older people^18^. People with many teeth consumed more of the majority of nutrients than those with fewer existing teeth^22^ and had a good dietary capability and optimum nutritional intake. The number of teeth and chewing ability are related to each other, as chewing difficulties due to tooth of loss may affect food diversity and dietary patterns^23^, which can lead to malnutrition^24^. In a cross-sectional study by Nishida et al.^25^ using US national health and nutrition survey data, low dietary calcium intake was found to affect periodontal disease growth. In addition, a previous case–control study also reported that calcium, riboflavin, and gingival health were significantly associated^26^. Calcium intake systemically affects the mineral density of the alveolar bone supporting the teeth and affects tooth retention. Dietary calcium also provides local enamel protection, which can prevent tooth loss due to caries^27^. In particular, the number of teeth and protein intake in the elderly are also related, which is likely to be related to mineral intake^28^.

Dietary changes due to the loss of teeth can cause not only periodontal disease but also various systemic diseases. It has been reported that the risk of chronic disease, such as cardiovascular disease and hypertension, increases with tooth loss that reduces fruit and vegetable intake in older age^13,29^. The consumption of foods such as fruits, vegetables and nuts has been reported to significantly reduce the risk of cardiovascular disease and stroke^30^. In addition, many studies have suggested that vegetarian food ingestion prevents cardiovascular disease^31^. Potassium is associated with the intake of vegetables and fruits; we found that as the number of existing teeth decreased, potassium intake decreased significantly (Table 2). Potassium plays an important role in the acid–base balance that affects dietary-related physiological functions, osteoporosis, aging, and hormonal action and is involved in the pH regulation of the intracellular organs, which plays an important role in the enzymatic regulation of metabolism in the human body^32^. High sodium intake and low potassium intake increase the sodium acidity of body fluids. As age increases, the risk of osteoporosis increases because of the reduced kidney function of releasing acids, using bases stored in bone or skeletal muscle^33^. In the adjusted model, the results of our study indicate that daily higher dietary calcium and potassium intake showed a significantly positive relationship with the number of existing permanent teeth in elderly Koreans. Only one previous study assessed the relationship between potassium and the number of existing permanent teeth in elderly Koreans^34^. The results of the preceding studies supported our findings. However, our data cannot compare with those studies due to the absence of information linking the number of teeth and dietary intake by using adjusted odds ratio models. The number of existing permanent teeth showed a negative correlation with carbohydrate and fat intake and a positive correlation with potassium intake.

This study has some limitations. First, we could not consider the parameters of household composition that could affect dietary status. Second, when estimating the number of existing permanent teeth, prosthetic status was not considered. And cross-sectional studies such as KNHANES are difficult to confirm causality and that there is no choice but to confirm the association. To tease out the role of nutrient intake and the number of teeth, better-designed studies will be needed.

Despite these limitations, this study also has major strengths. First, we used data from the KNHANES, which is a large, representative nationwide survey, wherein the participants were stratified using a multi-stage probability sampling design. To the best of our knowledge, the present study includes the largest sample size among studies focusing on potassium intake and bone metabolism. Second, qualified interviewers evaluated nutrient intake using a food frequency questionnaire, and nutritionists calculated the results. Therefore, a better way to assess the long-term effects of potassium intake on oral health would be to accurately estimate an individual’s daily potassium intake in the future studies.

## Conclusion

Low potassium intake is positively associated with tooth loss, indicating the beneficial effects of dietary potassium intake on oral health.
